# Resistance hysteresis correlated with synchrotron radiation surface studies in atomic sp^2^ layers of carbon synthesized on ferroelectric (001) lead zirconate titanate in an ultrahigh vacuum†

**DOI:** 10.1039/c9ra09131a

**Published:** 2020-01-08

**Authors:** Nicoleta Georgiana Apostol, Daniel Lizzit, George Adrian Lungu, Paolo Lacovig, Cristina Florentina Chirilă, Lucian Pintilie, Silvano Lizzit, Cristian Mihai Teodorescu

**Affiliations:** National Institute of Materials Physics Atomiştilor 405A, 077125 Măgurele – Ilfov Romania teodorescu@infim.ro; Elettra Sincrotrone Trieste Strada Statale 14 – km 163, 5, Area Science Park, 34149 Basovizza Trieste Italy

## Abstract

Carbon layers are deposited on 100 nm thick atomically clean (001) lead zirconate titanate (PZT) in ultrahigh vacuum, ruling out the presence of any contaminants. X-ray photoelectron spectroscopy is used to assess the substrate surface or interface composition, substrate polarization and the thickness of carbon layers, which ranges from less than one monolayer (1 ML) of graphene to several monolayers. Atomically clean PZT(001) exhibit inwards polarization, and this polarization reverses the sign upon carbon deposition. Cationic vacancies are detected near the PZT surface, consistent with heavy p doping of these films near the surface. The carbon layers exhibited a consistent proportion of atoms forming in-plane sp^2^ bonds, as detected by near-edge absorption fine structure (NEXAFS) analysis and confirmed partially by scanning tunneling microscopy (STM). *In situ* poling with simultaneous in-plane transport measurements revealed the presence of resistance anti-hysteresis *versus* the polarization orientation for films with less than 1 ML carbon amount, evolving towards ‘normal’ hysteresis for thicker carbon films. The anti-hysteresis is explained in terms of a mixed screening mechanism, involving charge carriers from the sp^2^ carbon layers together with holes or ionized acceptors in PZT(001) near the interface. For thicker films, the compensation mechanism becomes extrinsic, involving mostly electrons and holes from carbon, yielding the expected hysteresis.

## Abstract 2

Graphene-like layers synthesized in ultrahigh vacuum, characterized by surface science techniques, exhibit resistance hysteresis depending on the carbon coverage.

## Graphical abstract

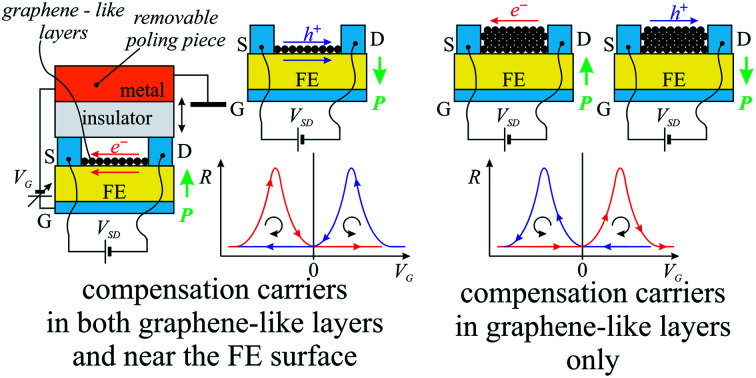

## Introduction

**Fig. 1:**
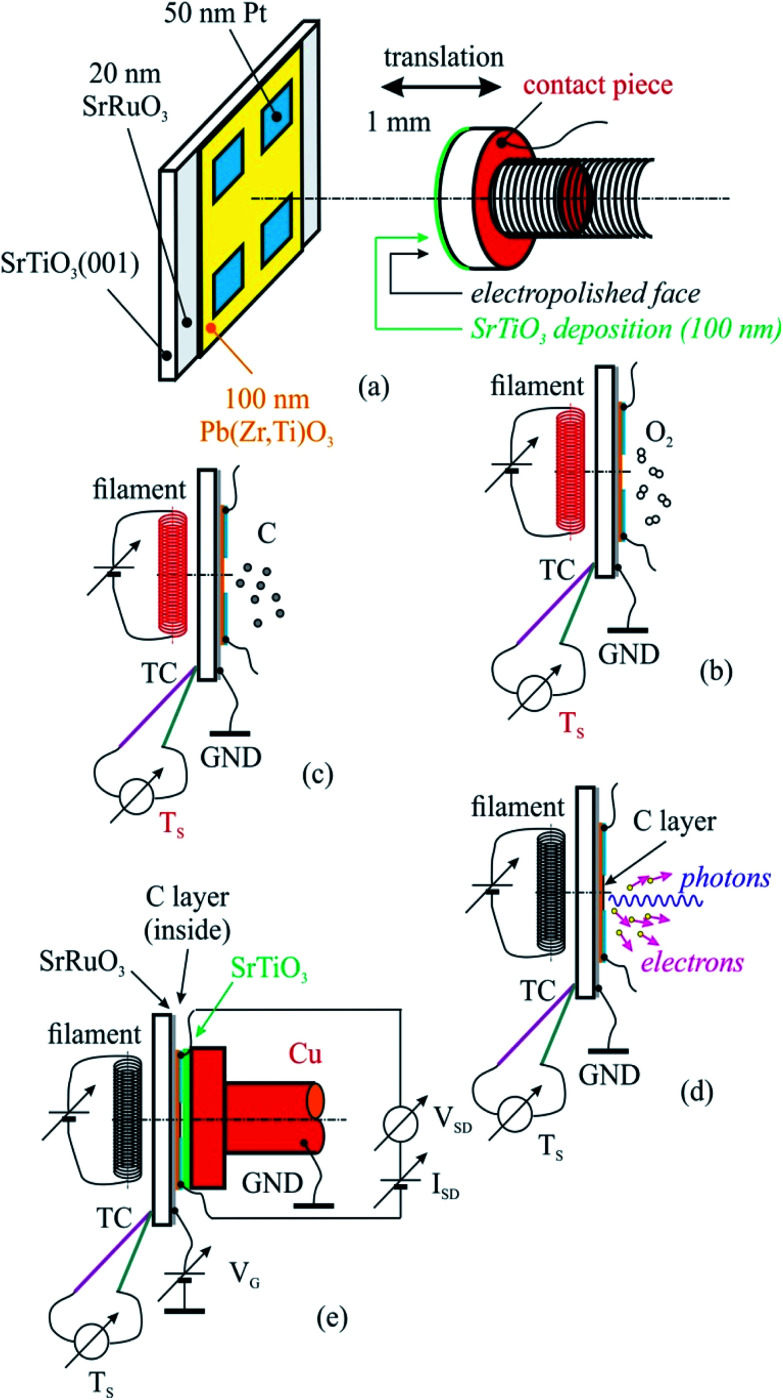
The sketch of the created heterostructure together with a poling piece (a) used for sample cleaning in ultrahigh vacuum (b), carbon deposition (c), correlated X-ray photoelectron spectroscopy (d), X-ray absorption spectroscopy (d), and electrical measurements (e).

Consistent efforts have been dedicated over the last few years to the coupling of graphene (Gr), graphene-like carbon films or other 2D films with ferroelectric materials, owing to the possibility to control the in-plane resistance of these films *via* the ferroelectric out-of-plane polarization of the substrates.^1–4^ In addition to the utilization of this effect for easy readable ultrafast non-volatile memories,^1,3^ due to the considerable dielectric constants, especially near the transition temperature, ferroelectric gates are actively investigated also for inducing high levels of electrostatic doping while using a low gate voltage, as compared to gating using ‘traditional’ SiO_2_. One key concept for the operation of such devices is charge compensation of the uniform depolarization field occurring in a ferroelectric thin film with out-of-plane polarization. In principle, the out-of-plane polarization of a ferroelectric film may be compensated by charge carriers from graphene or graphene-like layers by ‘extrinsic compensation’. The interest to use graphene stems in its zero band gap semiconducting character, allowing the conduction to be tuned continuously from n to p character, and to the high mobility of these charge carriers, allowing one to imagine rapid reading of resistance changes induced by different polarization states. Also, several types of chemical sensors may be imagined based on these heterostructures.^5^ By sweeping the gate voltage of a Gr on PZT based device (see Fig. 1(e) for a sketch of the device and Fig. 1 from ref. 6), one expects the conductivity of the graphene to exhibit the so called “normal” hysteresis.^6–8^ In more detail, assuming an ideal case of graphene on PZT in absence of interface and oxide traps, and with no graphene chemical doping, in correspondence to a change of the PZT polarization (from inwards to outwards and the *vice versa*) the Fermi level is aligned with the Dirac point leading to minima in the graphene conductivity. In particular, when the gate voltage *V*_G_ between the surface in contact with the graphene sheet and the back electrode of the ferroelectric film is increased towards positive values, *i.e.* forcing an outwards polarization (*P*^(+)^), the graphene Dirac point lies below the Fermi level leading to an increase in the conductivity which tends to saturate for increasing *V*_G_ values and where majority carriers are electrons. Then, by decreasing *V*_G_, the conductivity decreases up to a minimum which corresponds to the change of the PZT polarization (from outwards to inwards). Below this voltage the PZT polarization becomes inwards (*P*^(−)^) with the Dirac point lying above the Fermi level, thus leading to a new increase of the graphene conductivity given by hole conduction. Finally, by increasing again the gate voltage towards positive values, there will be a new minimum in the graphene conductivity when switching from *P*^(−)^ to *P*^(+)^ polarization. When the graphene layers exhibit an initial doping (in most cases of p type),^9^ the hysteresis occurs between two stable states with different surface conductivities,^9–13^ which can be viewed as switchable memory states (see *e.g.* Fig. 5 from ref. 13).

However, the first experiments with transferred graphene onto ferroelectric substrates revealed ‘anti-hysteretic’ behaviour, *i.e.* when the initial positive *V*_G_ is decreased towards zero, one records firstly a minimum in the conductivity (resistance maximum), then the conductivity is constant in the range of negative *V*_G_; then, when *V*_G_ is increased back to zero, the conductivity first exhibits a minimum (maximum resistance) in the range of negative *V*_G_, then it stays more or less constant in the range of positive *V*_G_.^14,15^ Explanations were attempted invoking several sources of external charges (“extrinsic” compensation), firstly involving dissociation/recombination dynamics of water molecules^14^ or trapping at interface states.^15^ Other explanations of the anti-hysteresis involved “relatively slow interactions with surrounding molecules such as H_2_O and O_2_, charge injection into interfacial trap states, and/or charge redistribution within the substrate caused by mobile and trapped charges”, “polarization screening from interface adsorbates and charge dynamic trapping/detrapping into the interface defect states”^16^ or possibly a dielectric layer formed by contaminants between graphene and the ferroelectric substrate.^17^ Transition from anti-hysteresis to hysteresis was reported by chemical etching^18^ or lowering of temperature.^19^ In the first case, this transition was explained by electrolyte insertion between graphene and the ferroelectric “compensating for anti-ferroelectric effects”, while in the second it was “attributed to the dynamic response of interface screening charges that are activated by the combined effects of electric field and temperature”. It was then recognized that working in cleaner conditions is needed and a few years ago experiments were reported in vacuum in the range of 10^−3^ to 10^−4^ Pa.^20–22^ Other precautions in terms of cleanness were undertaken, including thermal treatment.^1,23,24^ However, the results were not concluding in all cases and it was acknowledged that even working in the above vacuum conditions, the influence of chemisorbed OH^−^ and H^+^ cannot be ruled out.^22^

The charge carrier density in 1 ML graphene is insufficient to screen a polarization exceeding a few tenths of C m^−2^,^2,14^ see also the evaluation a few paragraphs below. In addition to the above mentioned hypotheses, other extrinsic compensation mechanisms were proposed, such as charge transfer driven by electronegativity differences between outermost atoms from ferroelectrics and carbon,^25^ direct interaction of graphene with defects, which modify carbon hybridization from sp^2^ to sp^3^ or charge injection into interfacial traps,^1^ dynamic accumulation of oxygen vacancies near the interface, forming a layer with different dielectric constant,^26^ charge trapping prevailing over ferroelectric effects,^6^ “intrinsic bulk ferroelectric hysteresis in combination of an oxygen-deficient surface layer”,^20^ O_2_/H_2_O redox couple intermediated by graphene charging.^24^ In the case of other 2D systems (*e.g.* hexagonal boron nitride, h-BN) used to separate graphene from the ferroelectric substrate, the resistance antihysteresis is explained in terms of the graphene conductance governed by the voltage on h-BN, which is derived from *V*_G_ by subtracting the voltage on the ferroelectric. When the latter exhibits hysteresis, then the voltage on h-BN exhibits anti-hysteresis.^12^ Note that hysteresis was obtained also in the case of use of non-ferroelectric separating material, SrTiO_3_,^27^ though more recent work did not confirm it.^28^ Using a more complex device involving the additional gating using a SiO_2_ layer one is able to switch between resistance hysteresis and asymmetric anti-hysteresis using this gate voltage.^29^ Note also that, even without a clear understanding of the diverse origins of charge compensation, most efforts to build up heterostructures with good endurance and fatigue results were successful.^6,21,24,29^

Theoretical efforts, mostly phenomenological, to simulate these behaviours take into account ferroelectric polarization, dipoles on the graphene and interface traps, together with switching times and dipole relaxation times,^7^ solve Laplace-like equations together with Landau–Ginsburg–Devonshire-type Euler–Lagrange equation for a system including a top oxide, graphene, a dead layer and a ferroelectric with domain walls,^8^ propose a piezoelectric mechanism of conductance control in the graphene field effect transistors on a domain structured ferroelectric substrate,^30^ including its temperature dependence.^31^ Density functional calculations on graphene deposited on lithium niobate proposed surface charge passivation by surface chemical reconstruction, *i.e.* the presence of additional Li ions on *P*^(+)^ surface, or additional O and Li on *P*^(−)^ surface. Charge compensation is achieved by LiNbO_3_ itself at 473 K, without contribution from graphene. Cooling down increases the polarization, without possible further compensation allowed only from the ferroelectric material, thus this compensation is needed from the graphene layers.^9^ This was one of the few references taking into account extra compensation of the ferroelectric polarization originating from the ferroelectric material itself.

In absence of any source of extrinsic compensation the huge depolarization field is expected to destroy the ferroelectric state, being larger than the coercive field of the material and oriented opposite to its bulk polarization.^32–35^ However ferroelectric thin films still exhibit a well defined out-of-plane polarization imprint. It was then proposed an intrinsic compensation mechanism provided by charge carriers in the ferroelectric film, which are separated towards the extremities of the film and accumulated such as to compensate the depolarization field: electrons near the face with polarization oriented outwards *P*^(+)^, holes near the face with inwards polarization *P*^(−)^.^32,33^ For ultrathin films, the usual doping levels in the ferroelectric semiconductor does not suffice to provide enough surface density of accumulated charge carriers and hence the material undergoes a “self-doping” phenomenon to generate enough charge carriers for compensation.^34^ Moreover, in the case of strong doping, the Fermi level would be placed near the conduction or the valence band and a compensation mechanism may be proposed also by a depleted layer of ionized impurities.^35^ If the thin film exhibiting out-of-plane polarization is strongly n-doped, then near the outwards polarized face electrons may accumulate, but near the inwards polarized face a layer of positively ionized donors may be formed instead of a layer of holes. Conversely, for a p-doped material, near the *P*^(+)^ face a depleted layer of negatively ionized donors may compensate the fixed positive polarization charges, and near the *P*^(−)^ face a layer of holes may be formed. These compensation mechanisms should still prevail or at least be non-negligible for graphene layers unable to compensate for polarization of more than a few tenths of C m^−2^.

Indeed, an immediate estimate may start with the dispersion law of graphene near one K point in the Brillouin zone (with wavevector ***k***_0_) *ε*(***k***) = *ℏc*_g_|***k*** − ***k***_0_|, then deriving the density of states with the usual formalism from the theory of free electron gases. One obtains the surface charge carrier density close to one of the six Dirac cones, assuming for the sake of simplicity a step-like Fermi–Dirac function:1
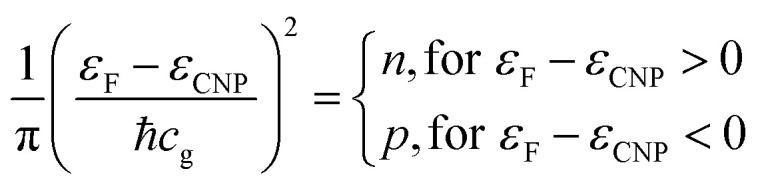
where *ε*_F_ is the Fermi energy and *ε*_CNP_ is the energy of the charge neutrality point (Dirac point), *ℏ* is the Planck constant and *c*_g_ the pseudo-light speed of the graphene ‘massless Dirac fermions’ (on the order of 10^6^ m s^−1^). Simulating the ‘exact’ dispersion law (see eqn (6) from ref. 36), a Dirac cone does not extend in energy further than ∼2 eV; thus, assuming the difference between the Fermi energy and the Dirac point in this range, a maximum surface charge compensation of about 0.47 C m^−2^ is obtained, in line with the estimate from ref. 2 of 8 × 10^17^ m^−2^ for 1 eV energy difference. Note that most surface band bending near the ferroelectric surface are in the range of 1 eV for a ferroelectric with about 1 C m^−2^ out-of-plane polarization.^37–40^ Thus, for a ferroelectric with larger polarization, such as lead zirconate titanate Pb(Zr,Ti)O_3_ (PZT) with more than 1 C m^−2^,^34^ in absence of any other extrinsic sources of charges (adsorbates, traps, *etc.*) the intrinsic compensation mechanism have to be taken into account in addition to compensation from graphene, otherwise, again, the residual depolarization field in the material may easily exceed its coercive field. This paper will interpret the observed anti-hysteresis in the case of ultrathin carbon layers in the framework of these considerations.

A second remark concerns the need to eliminate any other sources of extrinsic (rather uncontrolled) compensation mechanisms, which points to the need to use ultrahigh vacuum (UHV) during syntheses and characterizations. A simple estimate of the contamination rate from a residual gas yields a few seconds in a base pressure of 10^−4^ Pa.^41^ To our knowledge, no other previous investigations on such systems in UHV were performed so far. For example, free ferroelectric surfaces (without any contaminants or metal layers deposited on them), exhibit *P*^(−)^ state,^35,42,43^ despite the fact that most previous investigations reported a *P*^(+)^ polarization of thin PZT(001) films grown on SrRuO_3_/SrTiO_3_(001).^34,37–39^ This was explained through a complex mechanism driven by the build-in field at the bottom interface which promotes the inwards orientation of the polarization together with the p-type self-doping of the ferroelectric film.^35^

Next, surface or interface sensitive characterization tools must be used to assess both the graphene layers, the presence of adsorbates or other trapping states, and the ferroelectric film itself. Previous works mostly presented Raman spectra of graphene^9,12,17,26,27^ or atomic force microscopy (AFM), sometimes with piezoresponse force microscopy (PFM) characterizations.^3,6,10–12,14,18,21,23,24,27–29^ No surface chemical characterization in UHV such as (X-ray) photoelectron spectroscopy (XPS, PES) or X-ray absorption spectroscopy was reported previously on these kind of heterostructure. Photoelectron spectroscopy is able to derive the surface composition of the substrate, the chemical states of the different atomic species and, moreover, is able also to assess the surface polarization through a model implying intrinsic or extrinsic charge compensation.^34,35,37,38,42–45^ The asymmetry parameter of the C 1s spectrum recorded with high resolution is also a fingerprint of graphene-like structure.^13,46,47^ Near-edge absorption spectroscopy (NEXAFS) with angular resolution is able to assess the proportion of in-plane sp^2^ bonds, thus to give a global fingerprint of carbon organized in graphene-like layers.^46–49^ Scanning tunnelling microscopy (STM) can be performed with atomic resolution to give an immediate account on the atomic quality of the graphene layers. To these UHV and synchrotron-radiation based methods, we added the possibility to measure *in situ* on the films synthesized in UHV the resistance dependence on the applied gate voltage.

## Experimental details

Sample synthesis on ferroelectric oxide substrates by carbon molecular beam epitaxy^50,51^ is challenging since one needs to heat the substrates during carbon deposition to enhance the carbon mobility on the surface, but heating these substrates at temperatures above 900 K promotes their surface decomposition. Thus, a compromise had to be realized between the quality of the would-be graphene layers and the prevalence of the stoichiometry of the substrate. As the STM images did not show clear long-range ordered graphene, in this work we will not employ the word “graphene” for the samples synthesized and analysed, but rather “carbon films with in-plane sp^2^ bonding” (Csp^2^). However, in spite of these imperfections, we will suppose that the compensation mechanisms of the depolarization field are basically the same as for nearly perfect graphene overlayers; moreover, one of the main conclusions stemming in the insufficient amount of surface charge density in the graphene layer to compensate a strong polarization should be valid also for imperfect graphene or Csp^2^.

Note also that the deposition temperature chosen (above 800 K) is above the phase transition between the ferroelectric tetragonal and the paraelectric cubic states for Zr/(Ti + Zr) content below 50%.^52^ In other words, Csp^2^ is synthesized most probably on a film in paraelectric state. We will suppose in the following that the structure and morphology of the carbon film does not change significantly when the sample is cooled down and also that the extrinsic compensation mechanism occurs as soon the substrate becomes ferroelectric. It is not relevant to compare the results obtained by the actual synthesis procedure with similar experiments performed by depositing carbon at lower substrate temperature, where the ferroelectric state still subsists, since the morphology of the carbon layer obtained will differ significantly from that of a 2D would-be graphene, mainly due to the limited surface diffusion of carbon.

100 nm thick PZT/(20 nm) SrRuO_3_/SrTiO_3_(001) films were prepared by pulsed laser deposition (PLD) starting from a PbZr_0.2_Ti_0.8_O_3_ target enriched with PbO.^37,53^ X-ray absorption and photoemission experiments, STM and electrical measurements were performed on the SuperESCA beamline (including also the CoSMoS – Combined Spectroscopy and Microscopy on Surfaces – facility) at the synchrotron radiation facility Elettra, Trieste, Italy. More specifically, synchrotron radiation PES, NEXAFS and in-plane conduction experiments were performed in the analysis chamber of SuperESCA, while laboratory XPS and STM were performed on a similar sample in a different experiment in CoSMoS. The conventional XPS spectra are presented in the ESI.† The photon energies for the synchrotron radiation PES of the core levels of interest (260 eV for valence band, Pb 4f and Zr 3d, 600 eV for O 1s and Ti 2p, and 400 eV for C 1s) were chosen in order to record spectra with similar kinetic energies and surface sensitivity. The base pressure of the analysis chambers were in the 10^−9^ to low 10^−8^ Pa vacuum range. The electron energy analyzer was operated in “medium area” mode with pass energy 10 eV for core levels from the substrate and 5 eV for C 1s and photoelectrons are collected at 40° take-off angle. The beam size on the sample was 100 × 10 μm^2^ (width × height) and the photon flux at maximum beam intensity (400 eV photon energy) is estimated as 10^12^ s^−1^. NEXAFS spectra are measured by collecting C KLL Auger electrons and varying the incoming photon energy across the C K-edge together with the incidence angle of the linearly polarized beam on the surface.^47^ Scanning tunneling microscopy is performed by using STM 150 Aarhus system at room temperature in the constant current mode, *i.e.* by varying the approaching voltage of the tip (*V*_z_) such as to get a constant tunneling current and record maps of *V*_z_*vs.* the in-plane coordinates *x* and *y*.

The PZT(001) thin films were prepared such as they do not fully cover the strontium ruthenate (SRO) metallic substrate (for electrical contact reasons), and on each PZT(001) surface four 50 nm thick platinum contacts have been deposited by magnetron sputtering. In Fig. 1 we sketched the created heterostructure and all steps that we performed for cleaning, poling, C depositions and measurements.

For sample poling, a retractable device was used, consisting in a 5 mm diameter copper rod whose contact surface has been electropolished up to N1 level (*i.e.*, 25 nm roughness), and covered with a 100 nm thick insulating strontium titanate (STO) thin film deposited by PLD. The order of the operations in the present experiment was as follows:

(a) Introduction of the PZT/SRO/STO(001) sample with its electrical contacts in UHV chamber of the SuperESCA beamline setup, and its characterization *via* PES;

(b) Sample thermal cleaning at 400 °C in partial oxygen atmosphere (5 mPa pressure) during 4 hours, until the C 1s signal becomes negligible; chemical characterization by PES of the clean sample (core levels from all atoms from the substrate);

(c) Characterization of the sample by using electrical measurements, *i.e.*, bringing the poling device (grounded) in mechanical contact with sample surface (with electrical contacts), applying of a gate voltage between the SRO thin film and ground as well as applying of another voltage between two contacts on the sample surface, source and drain (*V*_SD_), measuring the in-plane electric current as function of the gate voltage *I*(*V*_G_). One thus obtains the electrical resistance *R*(*V*_G_) = *V*_SD_/*I*(*V*_G_). Conventionally, *V*_G_ > 0 stands for an electric field applied with positive (+) polarity on SRO and negative (−) polarity to ground, above the ferroelectric surface, *i.e.* poling the PZT(001) film outwards. The applied electric field is computed as *V*_G_/(100 nm STO + 50 nm free space + 100 nm PZT) ≈ 4 × 10^6^*V*_G_[V], being measured in V m^−1^. One performed the measurements in *V*_G_ = −5 V, …, +5 V ranges using Keithley electrometers with integrated sources for voltage output.

(d) Carbon deposition on annealed samples at 823 K, followed-up with ultrafast PES measurements (of the C 1s core level).

(e) Chemical characterization using PES (C 1s, Pb 4f, Zr 3d, Ti 2p, O 1s) of the prepared structure.

(f) Characterization using near edge X-ray absorption fine structure (NEXAFS) spectroscopy at carbon K edge for various incident angles of the prepared structure.

(g) Electrical measurements, according to procedure described in (c).

(h) Sample cleaning, according to procedure described in (b), and resuming the entire experiment for another carbon layer deposited.

## Results

**Fig. 2:**
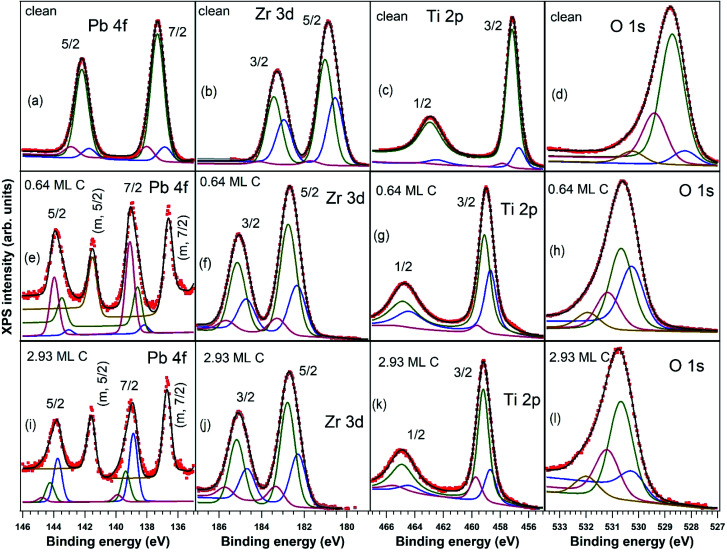
Deconvolutions of all core levels of interest (Pb 4f, Zr 3d, Ti 2p, O 1s) for the clean sample and for the two thinnest C depositions using Voigt profiles: (a, e and i) Pb 4f; (b, f and j) Zr 3d; (c, g and k) Ti 2p; (d, h and l) O 1s with (a–d) clean PZT(001), (e–h) 0.64 ML carbon deposited, (i–l) 2.93 ML carbon deposited.

**Fig. 3:**
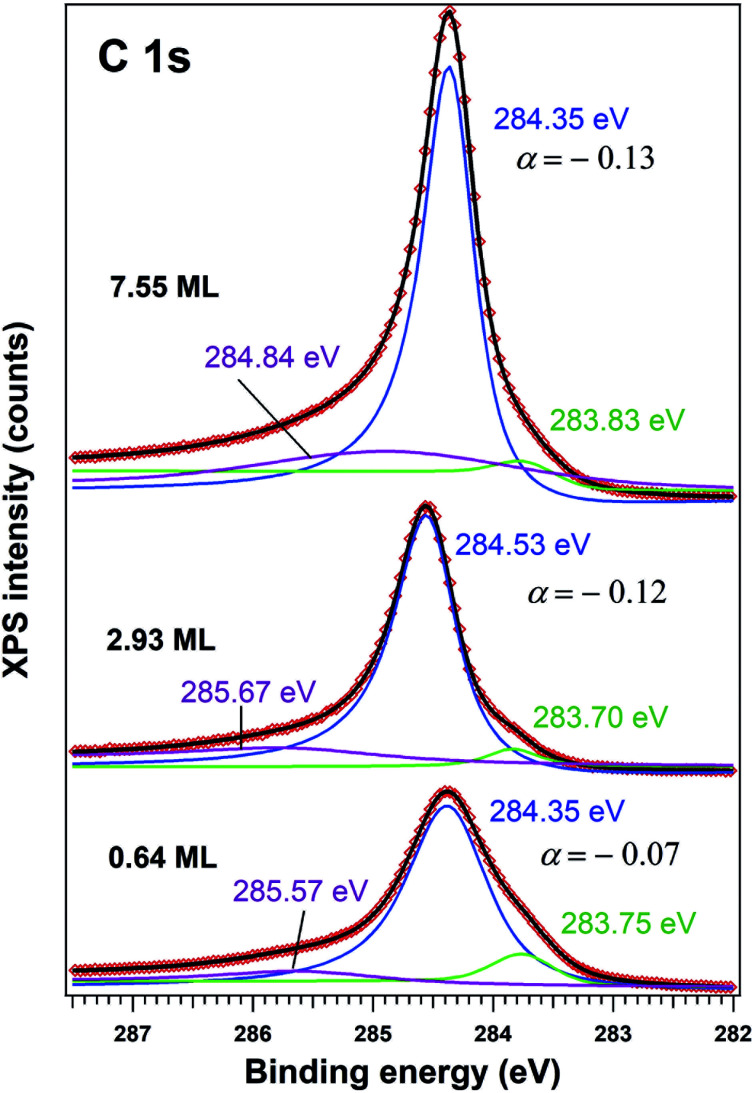
X-ray photoelectron spectra recorded for C 1s region (using 400 eV incident photons energy), deconvoluted using Doniach–Šunjić–Gauss profiles (the principal, blue lines), plus two symmetric lines (Voigt profiles, green and magenta coloured).

X-ray photoelectron spectroscopy measurements, with detailed spectra for Pb 4f, Zr 3d, Ti 2p, and O 1s core levels and their deconvolutions, are shown in Fig. 2, and C 1s core level photoelectron spectra are shown in Fig. 3. Using substrate core level recorded spectra, one inferred the substrate composition based on the analysis of integral amplitudes, as well as the ferroelectric polarization states, based on analysis of the core levels chemical shift. Owing to the different excitation energies for the various elements, in order to compute the atomic concentrations, one used the photoionization cross sections tabulated in ref. 54 as well as the internal calibration of the photon flux at different photon energies available at SuperESCA beamline. The preparations discussed in this work are indexed with the number of equivalent carbon monolayers (ML), 1 ML denoting a single graphene layer, corresponding to one C atom for 2.62 Å^2^. The carbon coverage was estimated from the attenuation of the core levels of the clean sample, assuming an exponential decay due to inelastic mean free path effects. This was also cross checked with the integral amplitude of the C 1s spectra related to integrals of core levels from the clean sample and, again, weighted by atomic photoionization cross sections and by the beam intensity. Note also that for single crystalline films intensity ratios may be biased from real values owing to photoelectron diffraction effects.^42^

All core levels of the ferroelectric layer were analysed using ‘deconvolutions’ with Voigt lines.^55,56^ In the present work we simultaneously fitted all X-ray photoelectron spectra (Pb, Zr, Ti, O) for a certain sample state, imposing, for instance, equal relative chemical shifts for all photoemission lines.^40^ The exceptions are: (i) the O 1s spectrum, where an additional line at higher binding energy (BE) had to be introduced; (ii) the Pb 4f spectrum, where an additional doublet at lower BE needed to be considered for samples with C deposition. This may be attributed to the formation of metallic Pb during carbon deposition.^45,57^

One also observes that, just after the carbon deposition, all core levels are shifted to larger binding energies with about 2 eV (less for Zr) and also the occurrence of narrow low BE peaks in the Pb spectra, attribute to the formation of metal Pb on the surface. The atomically clean sample evidenced an inward electric polarization *P*^(−)^, as in other experiments.^42^ After carbon deposition, the observed shift towards higher binding energies is too large to be assigned only to the band bending loss in the semiconductor, in the hypothesis that the depolarization charge would be localized only in graphene layers. Rather, this observation suggests that an opposite polarization is induced in PZT(001), *i.e.* an outwards polarization, *P*^(+)^. A possible effect of the pre-poling originating from the electrical measurements performed on the clean sample with different gate voltages may be neglected, since: (i) as stated above, the carbon deposition is performed at temperatures where the polarization is expected to vanish or, in any case, to decrease considerably; (ii) anyway, the electrical characterization of the clean sample was performed only after the first sample cleaning; the subsequent sample cleaning were characterized by PES to check cleanness, then C was deposited immediately, then again XPS and at the end electrical measurements. All results concerning atomic ratios and derived polarizations of PZT(001) are summarized in Table 1. For the clean sample, the ratio between the oxygen signal and all cations is close to 1.5; the relative increase in the Pb content may be attributed to inelastic mean free path effects, photoelectron diffraction or some PbO excess on the sample surface. One also observes from the chemical composition analysis that the PZT(001) film is highly depleted in Pb near the surface after carbon deposition, though initially the sample was rich in Pb at the surface. Note that from laboratory XPS experiments on a twin sample, presented in the ESI,† the Zr content was 0.21 instead of 0.33, so photoelectron diffraction cannot be excluded from the analysis of surface sensitive PES. These experiments also have shown Pb depletion when carbon is deposited, without any Pb reduction.

**Table 1:** Main results obtained from analysis of individual XP spectra and NEXAFS

Sample	Pb/(Zr + Ti)	Zr/(Zr + Ti)	O/(Zr + Ti)	C/(Zr + Ti)	C coverage	Polarization	*p*(sp^2^)
Annealed	1.74	0.33	4.09	0.05	N/A	Inwards	—
C deposition	0.28	0.32	3.12	3.74	0.63 ML	Outwards	0.638
	0.34	0.29	2.55	17.04	2.93 ML	Outwards	0.415
	Not measurable				7.55 ML	Outwards	0.003

The main signals from the substrate, with the exception of the metal Pb peak denoted with “m” in Fig. 2(e and i) and of the additional O 1s component, are simulated with a main peak and with two smaller components, one at a larger and one at a lower BE. Pb 4f spectra of nearly stoichiometric atomically clean PZT(001) thin films (10 nm), discussed in ref. 42, were simulated with a main component and a high BE component, the latter being ascribed to Pb from the surface PbO atomic layer, with lower oxygen coordination. A similar significance may have in the actual case all cationic high BE component (Pb, Zr and Ti spectra). This is a sign that the PZT film is not uniquely PbO or (Zr,Ti)O_2_ terminated. The line of lower intensity at lower BE may be attributed to atoms neighbouring other cationic vacancies, where the local Madelung-like potential is perturbed by a term yielding globally to a negative potential energy, with respect to the perfect crystal. Similar considerations may apply also for the O 1s spectrum: local oxygen deficiency near surface for the higher BE component, and local cationic defects for the lower BE component. The additional component at even higher BE (about 530 eV for the clean sample and 532 eV after C deposition) may be attributed *e.g.* to adsorbed hydroxyls on the surface.^58^ We stress again that this whole manifold of states, all originating from near surface atoms, shift with the polarization change, as seen readily from Fig. 2, and consistent with the model of intrinsic compensation, see *e.g.* Fig. 1 from ref. 35.

The C 1s spectra (Fig. 3) are deconvoluted with a Doniach–Šunjić lineshape combined with a Gaussian.^59^ The asymmetry parameter *α* for depositions exceeding 1 ML is in line with values derived for graphene films,^47,60^ while for ∼0.6 ML the asymmetry parameter is lower, with the occurrence of two other components, most probably related to carbon in different environments. The C 1s component at higher binding energy most probably is due to carbon near the interface cations from PZT(001) (maybe several carbon states are contributing to this broader component), while the component at low binding energy could be due to carbon sitting on oxygen-rich areas^61^ or to a carbide peak. As a consequence, carbon layers grown in this work do not represent single graphene layers, but rather carbon in graphene-like environments mixed with carbon bound to oxygen and to cations from the substrate.

**Fig. 4:**
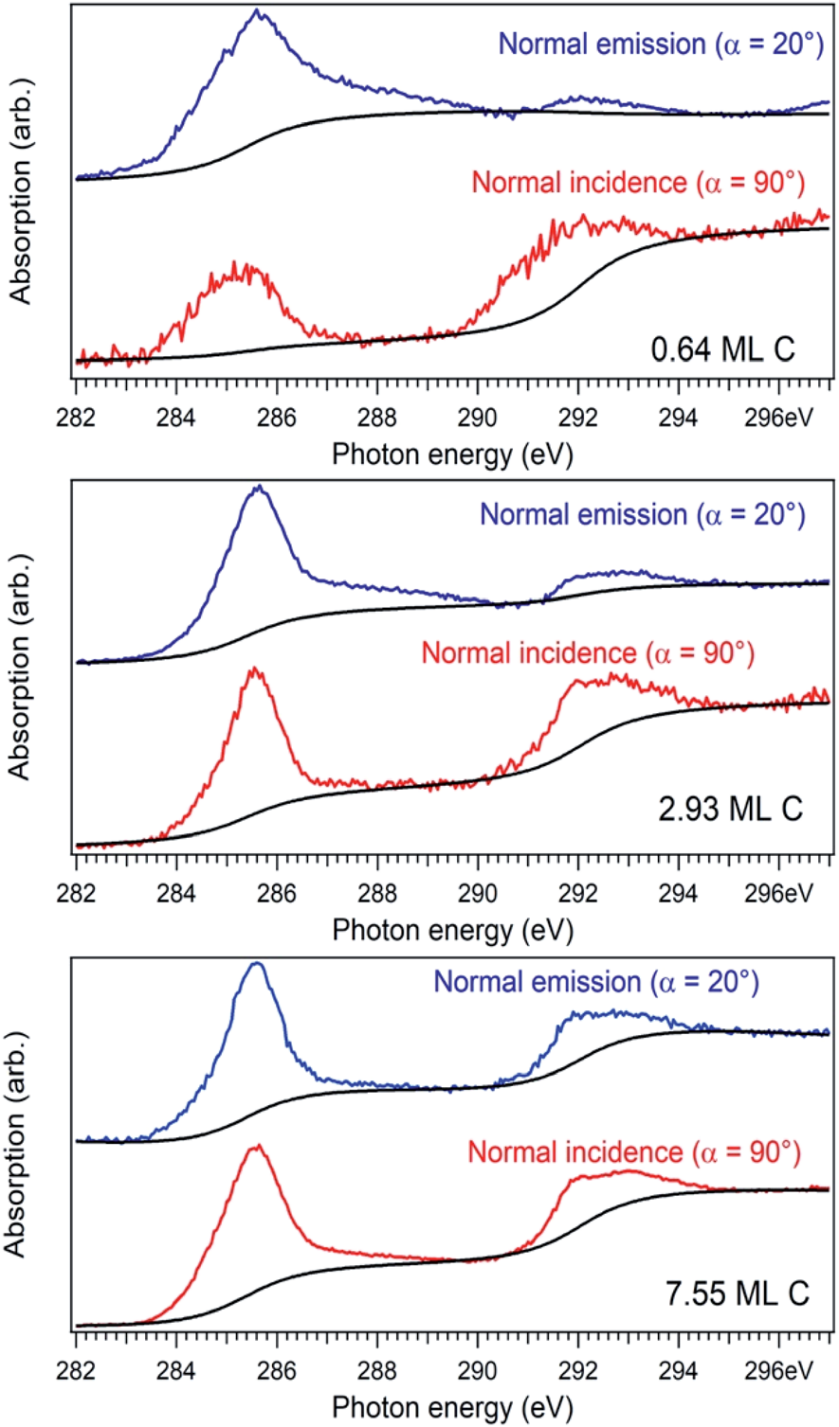
Near-edge absorption fine structure (NEXAFS) spectra for the carbon K edge in the case of normal X-ray incident beam (*α* = 90°, red curves) and glancing incident X-ray incident beam (*α* = 20°, blue curves).

For a better accounting of the amount of carbon forming in-plane sp^2^ bonds (constituting 2D seeds for graphene), the samples were analysed by near-edge X-ray absorption fine structure (NEXAFS) spectra at the carbon K edge, recorded for two different (normal and grazing) incidence angle of the X-ray beam with linear polarization. These spectra are represented in Fig. 4 and feature two main structures, attributed to transitions of C 1s electrons to unoccupied σ* and π* orbitals.^47–49^ We define the intensity for both σ* and π* transitions as the integral of the signal for the respective lines after the subtraction of a double arctangent background (Fig. 4, black curves). One can observe that for the thickest carbon deposited layer (7.55 ML), the dichroism (defined here as the difference of the signals for the two absorption spectra in normal and grazing incidence) almost vanishes. This is a sign that for the thickest layers the amount of carbon forming preferentially in-plane sp^2^ bonds becomes negligible.

On the contrary, the other two carbon coverages investigated exhibit a measurable dichroism in NEXAFS. One can then estimate the amount of in-plane sp^2^ bonds with a simple model, described in the following. If we denote by *φ* the angle between the X-ray beam polarization and the normal to the surface,^47^ the intensity of 1s → 2pσ* and 1s → 2pπ* transitions are described (from the evaluation of one-electron transition matrix elements from 1s to final p_*z*_ or p_*x*,*y*_ states) by:2
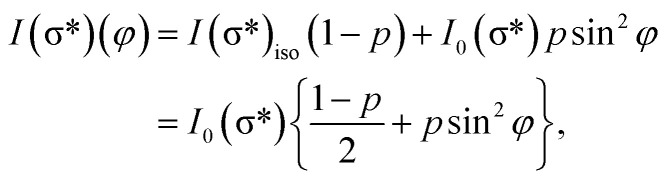
3
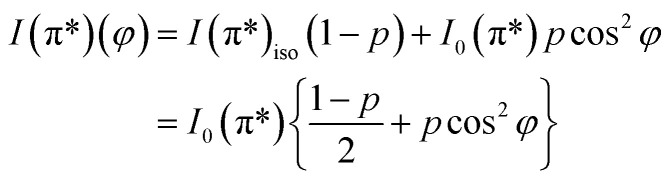
where *I*(σ*)_iso_ stands for the contribution to the signal from carbon atoms having randomly directed p atomic orbitals, which equals *I*_0_/2 if one considers the average of sin^2^ *φ* and cos^2^ *φ* for all directions. Thus, one can calculate the ratio between transitions to π* and σ* antibonding molecular orbitals:4
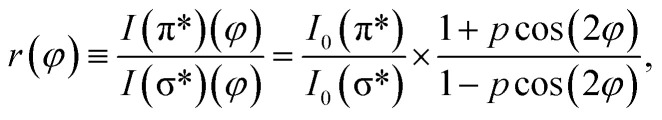


In the present case, introducing the used take-off angle values,5
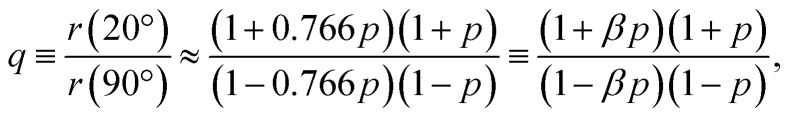
where *β* = cos 40° ≈ 0.766. Solving this equation for *p*:6
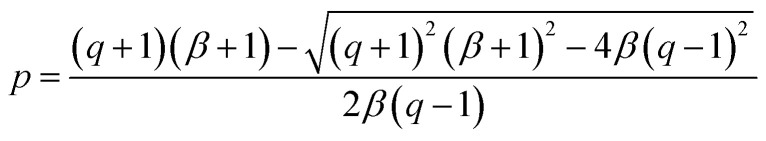


The values obtained for *p*(sp^2^) are also represented in Table 1. These proportions can be used to estimate the electric resistance of the long-range two-dimensional (2D) carbon structure (exhibiting plane sp^2^ hybridization), when the total electric resistance of layers is known, see the ESI.†

**Fig. 5:**
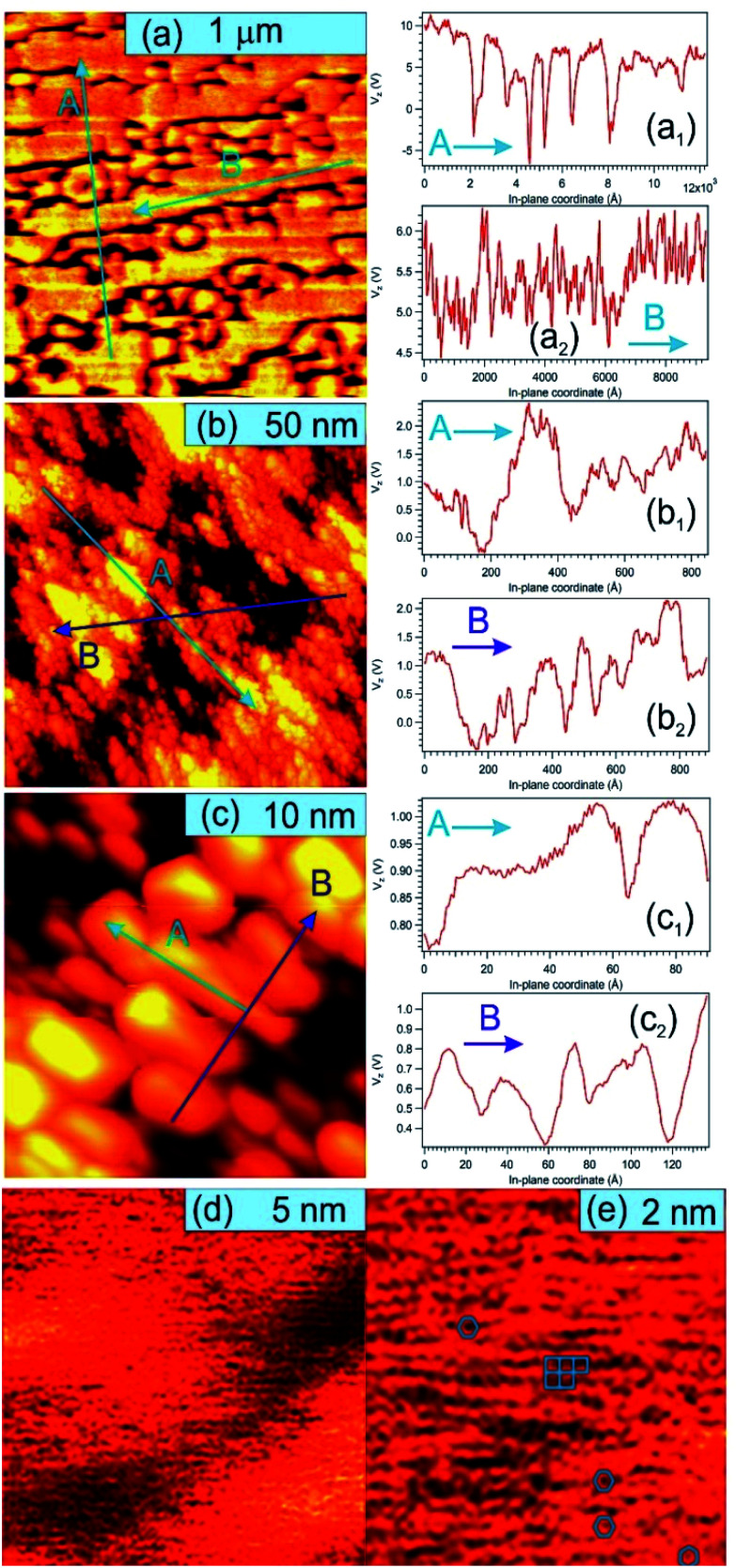
STM images for a carbon film of about 0.7 ML grown on PZT(001). The image sizes are (a) 1.5 μm, *V*_t_ = 1.25 V, *I*_t_ = 1.83 nA; (b) 100 nm, *V*_t_ = 1.25 V, *I*_t_ = 90 pA; (c) 20 nm, *V*_t_ = 1.25 V, *I*_t_ = 60 pA; (d) 10 nm, *V*_t_ = 5.93 V, *I*_t_ = 260 pA; (e) 5 nm, *V*_t_ = 1.27 V, *I*_t_ = 270 pA. For (a–c), the left image represents the STM images, and the right graphs represent line scans, traced as function of the tip control voltage along the normal direction to the sample plane *V*_z_, plotted along the directions denoted by A and B on the images. (d) and (e) were Fourier filtered. The blue bar on each image represent the designated scale.

Fig. 5 presents STM results obtained on a sample prepared separately in similar conditions, with C coverage in the range of 1 ML. On a macroscopic scale (Fig. 5(a)) one can remark elongated stripes separated by deep (∼15 nm) valleys. Since the flatness of the PZT(001) film was proven by a wealth of methods (see *e.g.*ref. 34, 35, 38 and 43) and the irregularities are at most a few atomic interplanar distances (2–6 Å), 15 nm deep valleys cannot be explained by the sample morphology on atomic scale. This observation can be related more to changes in the conduction properties of the surface (*e.g.* the long stripes represent graphene layers) or different polarization states of PZT(001). Indeed, the range of the tip approaching voltage variation (*V*_z_) of *ca.* 10–15 V between bright and dark regions from Fig. 5(a) is in the range of twice the outer potential of a ferroelectric PZT(001), as derived from low energy electron diffraction (LEED).^43^ For this reason, all profiles in Fig. 5 are traced as function of *V*_z_ variations instead of topographic height, where a conversion factor along *z* of 1.3 nm V^−1^ has to be taken into account. On nanoscopic scale (Fig. 5(b and c)) the stripes are composed by islands a few nm wide and a few Å high (or a few tenths of volts in terms of *V*_z_), most probably formed by one or two ML of carbon or, equivalently, by areas with different conduction properties manifesting in the distance needed to maintain the tip with respect to the surface for a constant tunnelling current. On atomic scale, we could not detect a long range hexagonal pattern, but rather a mixture between hexagonal local atomic arrangements and rectangular local order, ascribed to the geometry of the PZT(001) surface (Fig. 5(d and e)). Consequently, we may infer that locally some carbon is placed in hexagonal atomic arrangement, but there is no long range honeycomb structure. This fact that some carbon may be found in honeycomb local arrangement is in line with the NEXAFS observations. Once more, we stress on the assumption that the PZT(001) surface, with or without carbon deposited on it, is flat at the level of a few interplane distances, and the ‘topology’ observed on larger areas reflects the landscape of the polarization and/or local conduction inhomogeneity. The voltage on the vertical motion of the piezo motor (*V*_z_) and the height of the tip are also assumed to be in a linear dependence, if one assumes ‘normal’ piezoelectric effect. The relative energy positions of the tip and sample densities of states is assumed the same for a constant current. The main questionable assertion is that the DOS of the sample is shifting linearly with the polarization but, anyway, *V*_z_ variations seem to be connected more to local polarization and conduction properties than to the real topography of the sample.^62^ Some details about the linearity between variations of *V*_z_ needed to maintain a constant tunnelling current (δ*V*_z_) and the occurrence of some bias voltages on the surface (δ*V*_b_) are given in the ESI.† The proportionality constant is in the range of several units, but at this stage of knowledge of all parameters involved it is difficult to estimate this proportionality constant with better precision. This will be the subject of a more dedicated work, and some very preliminary evaluations will be given at the end of the next Section. The conclusion of this discussion is that there are good reasons to suppose that the structures observed in STM on mesoscopic scale might be related to areas with different polarization orientation.

**Fig. 6:**
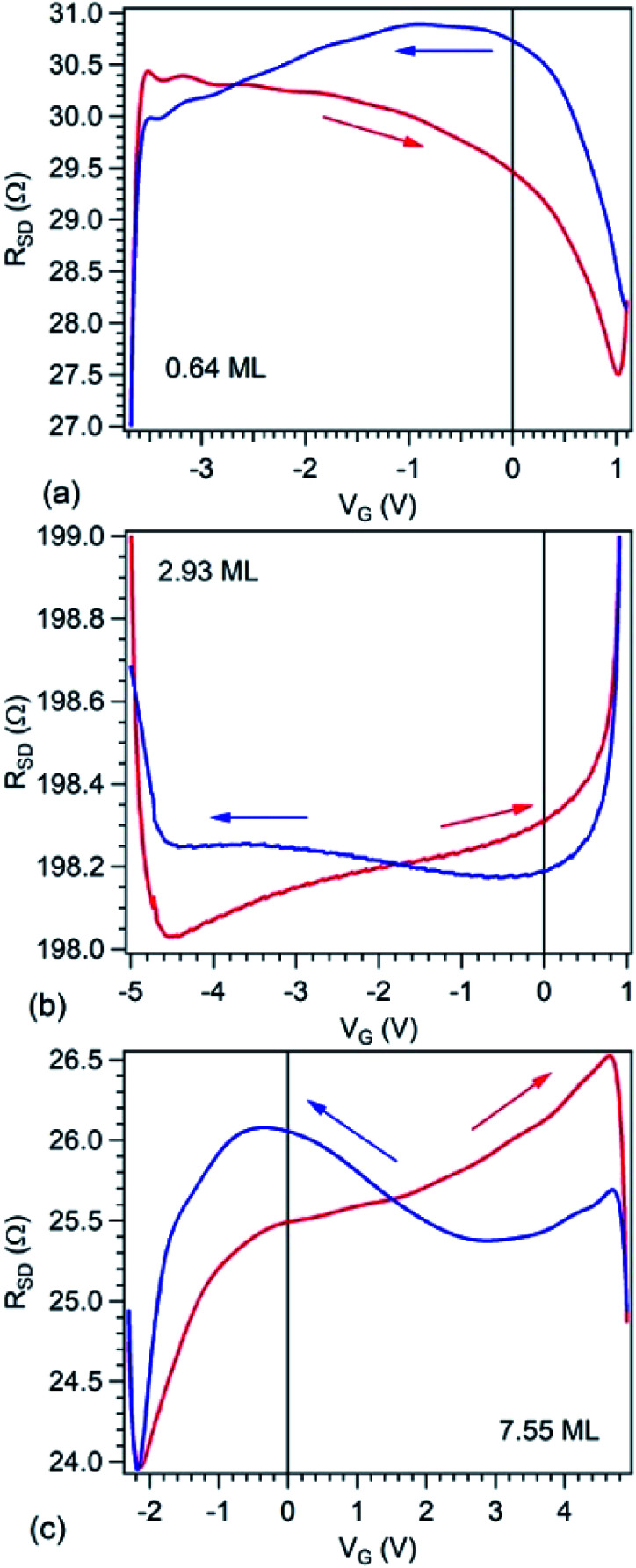
Plot of the electrical measurements, *i.e.* electric resistance between source and drain electrodes *R*_SD_ as function of applied gate voltage *V*_G_ on PZT(001) for the three films investigated by XPS and NEXAFS, in order of increasing carbon coverage, expressed on the graphs in terms of single graphene layers. The value of the contact resistance of 186.4 Ω was subtracted.

Fig. 6 presents source–drain resistance measured for different gate voltages. From the raw data *I*_SD_(*V*_G_) at a given *V*_SD_ (see Fig. 1(e)), a constant slope was subtracted, before calculating the resistances. This slope is most probably due to parasitic resistances or leaks through the STO film covering the copper poling piece. The obtained resistances are plotted *vs. V*_G_ for forward or backward scans, and resistance hysteresis is detected in all cases. (Note that a constant resistance attributed to contacts was subtracted, following an estimate which will be discussed in the next Section.) By comparing with the literature described in the Introduction, we may infer that in the case of the lowest carbon coverage (0.64 ML) anti-hysteresis is detected (for *V*_G_ scanned between *V*_max._ → −*V*_max._ the resistance is larger, exhibiting a local maximum before the middle of the scan, than when the cycle is covered from −*V*_max._ to *V*_max._, again, with a local maximum in this range before the middle of the scan); and in the case of thicker carbon films ‘normal’ hysteresis is detected. The occurrence of anti-hysteresis for the thinnest carbon film will be discussed in the following. We neglect the contribution of metal Pb generated by soft X-ray irradiation prior to electrical measurements since, if this phenomenon occurs only on a tiny spot of 10 × 100 μm^2^, this spatial region may be neglected with respect to the total area responsible for *R*(*V*_G_) measurement, whose size is above 1 mm^2^.

## Discussion

The main subject for this Section is the explanation of the transition from anti-hysteresis to hysteresis when the carbon coverage increases, but one has to address also the following aspects: (i) the switching of the PZT(001) polarization from *P*^(−)^ to *P*^(+)^ when carbon is deposited; (ii) the release of neutral (metal) Pb from PZT(001) when carbon is deposited; (iii) one has also to estimate the electrical parameters of the Csp^2^ film, and to estimate how close was it from the conduction point of view to ideal graphene.

Most PZT(001) films with thickness lower than 100 nm synthesized on SRO/STO(001), measured in air, *i.e.* contaminated, exhibited *P*^(+)^ polarization imprint, see *e.g.*ref. 34, 37, 38 and 63. In the Introduction it was stated that only when these films are prepared by cleaning in UHV they exhibit *P*^(−)^ polarization. This was explained by a mechanism taking into account the work function difference between PZT and SRO to create an interface field which initiate the polarization during the growth of the film (or its cooling down), together with the p-type self-doping of PZT(001).^35^ In the actual case, despite the uncertainties due to photoelectron diffraction effects and/or inaccurate evaluation of the photon flux, one obtains a (O : cations) ratio of almost 1.5 (so close to the ideal case), but larger than 2 for the cases with carbon deposited. Thus, probably the PZT(001) is nearly intrinsic near its outer surface (the p-type self-doping is weak and distributed in the whole thickness of the film); after carbon deposition at temperatures larger than the cleaning temperatures, cationic vacancies are present in a more significant amount near the outer surface. This p-type doping of PZT(001) implies that, in absence of extrinsic screening, a positive charge sheet is formed in PZT near the surface. When the doping is important, the top of the valence band and the acceptor levels are close enough (within a few tenths of eV), the positive charge layer will be formed by holes accumulated near the surface.

When carbon is deposited on this surface, its workfunction of 5.0 eV (ref. 64) is below that of p-type PZT(001), estimated at about 6.15 eV.^35^ Therefore, electrons will be injected from carbon to PZT(001), yielding a partial neutralization of the positive layer of holes near the outer surface of PZT(001). We argued in the Introduction that a single graphene layer is unable to provide enough charge carriers to compensate for a strong polarization such as that of PZT. However, looking to Fig. 5(a), carbon layers are continuous on a macroscopic extent and they could shunt the surface, providing electrons from outside. A similar mechanism was proposed for Cu *vs.* Au films on PZT(001).^39,63^ Thus, by carbon deposition, electrons are injected inside PZT(001), the positive (hole) layer is destroyed by recombination, and excess electrons near the surface together with holes from the volume induce the switch of the polarization, which becomes oriented outwards.

The release of the neutral Pb during C deposition may have two origins, both related to the high temperature needed during the carbon deposition. One hypothesis is that during this heating, the polarization of PZT(001) is not completely lost and Pb ion migrate towards the surface, then electrons provided by the incipient carbon films are neutralizing these ions. The second hypothesis involve the fact that carbon deposition was followed-up by PES, thus the sample at high temperature was continuously irradiated with a high intensity synchrotron radiation beam. Release of metal lead from PZT(001) subject to a high beam flux (∼5 × 10^11^ photons per (s μm^2^)) was reported and analysed in ref. 45. In the actual case, the beam flux is in the range of 10^9^ photons per (s μm^2^), but the photon energy is larger and several de-excitation channels with respect to that outlined from the above reference may contribute to the creation of ‘hot electrons’ which induce Pb–O surface dissociation. Note that the thinnest carbon film was still deposited in about 3 hours, whereas in ref. 45 significant surface photo-dissociation was recorded in a few (2–3) minutes. Thus, the ratio between the dissociation rates and the order of magnitude of beam fluxes are similar. Moreover, in the separate experiment where just 0.6 ML carbon were deposited without being analysed with the synchrotron radiation beam during the deposition, the laboratory XPS analysis using Al K_α_ monochromated X-ray source performed after the C deposition did not detect any dissociated Pb on the surface, see the ESI.†

**Fig. 7:**
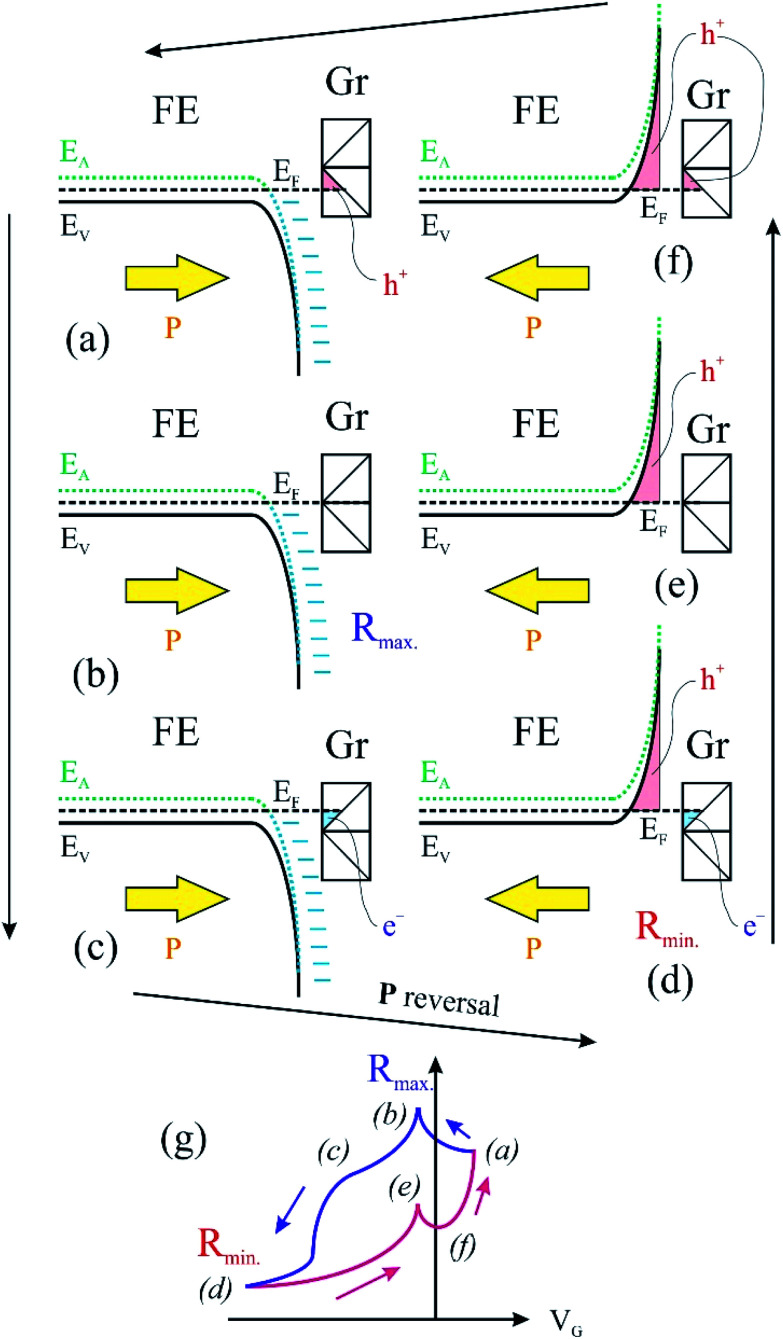
An intuitive model which considers charge carriers in both graphene and ferroelectric material, contributing to compensation of depolarizing field. FE is the ferroelectric layer; Gr represents the surface graphene. (a)–(f) represent several states during the ferroelectric hysteresis cycle and (g) a sketch of the variation of the in-plane resistance dependence of the gate voltage.

We turn now to the analysis of the fact that for the sample with 0.64 ML thick deposited carbon one obtains an inverse *R*(*V*_G_) hysteresis loop, while for both 2.93 ML and 7.55 ML the *R*(*V*_G_) dependence hysteresis loop is normally covered. We already mentioned several times that, below 1 ML carbon, the surface carrier density which could be provided by a graphene layer does not suffice to screen the depolarization field in PZT(001). Thus, a mixed intrinsic and extrinsic screening is to be expected in this case. We remind that the PZT(001) is p-doped. For outwards polarization, the mixed screening could be formed by electrons in graphene and negative acceptors in PZT, but in fact it was derived from the valence band spectra that also the Csp^2^ is p doped. Thus, the relaxed sample with outwards polarization should exhibit a low amount of charge in form of holes in the Csp^2^ and an important surface charge density in forms of negative acceptors near the interface. The situation is described in Fig. 7(a). When the gate voltage is decreased towards negative values, the hole density in Csp^2^ decreases, then the electron density starts to increase, and at the same time there are no mobile charge carriers in PZT(001), see Fig. 7(b). Consequently, the surface conductivity exhibits a minimum, and the resistance shows a maximum, the overall resistance being more elevated related to the other branch (increasing *V*_G_). This is also visible in Fig. 6(a). When, for *V*_G_ exceeding the negative coercive point, the polarization is reversed, the compensation charges in PZT(001) are holes, with an increase in the surface conductivity and a decrease of the resistivity, as represented in Fig. 7(d). Therefore, for the cycle *V*_G_ = – *V*_max._ → +*V*_max._ the resistance exhibit lower values than for the +*V*_max._ → −*V*_max._ (Fig. 7(d–f)). A sketch of the *R*(*V*_G_) dependence is represented in Fig. 7(g) and this is qualitatively in agreement with Fig. 6(a).

In this case, a measurement of in-plane conduction properties without any carbon deposited should also exhibit anti-hysteresis with higher resistance in the region where the film exhibits outwards polarization (compensation ensured by fixed negatively ionized acceptors) and lower resistance in the region of inwards polarization (compensation ensured by mobile holes accumulated near surface). Indeed, this was observed for a clean sample, but the measurements are much noisier than the ones represented in Fig. 6, so we did not represent them.

For Csp^2^ layers exceeding the equivalent of 2 ML of graphene, it seems that their surface charge density becomes sufficient to screen the depolarization field. In this case, one may suppose that the screening becomes extrinsic, and a ‘normal’ hysteresis cycle is obtained.^6^ The problem is that the valence band spectra of films with higher carbon coverage, represented in Fig. S3,† can still be interpreted in terms of p-doping of the Csp^2^. The resistance hysteresis in the case of 2.93 ML graphene is quite low, so we suggest that this thickness is close to the transition from compensation by ionized acceptors and compensation by electrons in graphene for the *P*^(+)^ polarized state. But in the case of the 7.55 ML film, the hole doping decreases significantly (Fig. S3†) and one has to take into account that only the outer carbon layers are investigated by valence band photoemission (the estimated inelastic mean free path is about 7 Å). Thus, it is possible that the outer carbon layers, giving the main contribution to the valence band photoemission spectrum are still slightly p doped, while the inner carbon layers should contain excess electrons to screen the depolarization film for the *P*^(+)^ state of PZT(001).

The order of magnitude of voltages where the resistance hysteresis manifests should be on the order of local voltage biases (δ*V*_b_), therefore in the range of 2–4 V. By comparing with the (δ*V*_z_) variations from the STM analysis on mesoscopic scale (Fig. 5(b and c)), one infers a factor of 5–10 between (δ*V*_b_) and (δ*V*_z_), which is in line with the simulations presented in the ESI.† But an additional factor must be taken into account. In fact, from the gate voltage only a fraction of about {1 + *t*_0_*ε*_PZT_/*t*_PZT_}^−1^ represents the voltage on the PZT(001) film, where *t*_PZT_ is the thickness of the PZT film, *t*_0_ the thickness of the free space (see the Experimental part) and *ε*_PZT_ is the dielectric constant of PZT. (The dielectric constant of the STO film deposited on the poling piece was considered very large, about 300.^65^) It is difficult to estimate both *t*_0_ and *ε*_PZT_. The first one is in principle 50 nm, but due to the roughness of the poling piece and of the deposited STO film an ‘effective thickness’ of half of the above value could be easily taken into account. The dielectric constant of PZT is also difficult to be estimated, but it can be as low as 30 (ref. 43) or even between 10 and 15.^66^ In total, it might be that the voltage drop on PZT could be 0.2–0.3 from the measured gate voltage in Fig. 6. Thus, the proportionality factor between the typical values needed to reverse the polarization, assumed on the order of (δ*V*_b_), and (δ*V*_z_) is reduced to 1–2, which is still acceptable in view of the simulations presented in the ESI,† but suppose larger tip–sample distances during the STM scan.

## Conclusions

This paper comments on the hysteretic properties of in-plane conduction of carbon layers with a significant proportion of in-plane sp^2^ bonds, as function of the polarization of a ferroelectric substrate, the whole experiment being conducted in ultrahigh vacuum, thus in absence of contaminants invoked in the past to explain unusual hysteretic behaviours. These measurements are correlated with extensive characterization by surface science tools of the clean surfaces and of the heterostructures formed, by photoelectron spectroscopy, scanning tunnelling microscopy and near-edge absorption fine structure. Despite the fact that STM did not show a long-range graphene-like order but rather a local organization with in-plane sp^2^ bonding (confirmed also by NEXAFS), the carrier mobility in the carbon layers is reasonable and may be compared with most reports on (imperfect) transferred graphene films. The absolute value of resistance asymmetry is quite low (Δ*R* is at most 1 Ω when the *V*_G_ is cycled), so applications cannot be foreseen starting with such films synthesized in UHV; nevertheless, the actual results obtained by ruling out external contaminating molecules or radicals contribute to a better understanding of compensation mechanisms which may affect the hysteretic behaviour. Other effects evidenced during this study are the change of the polarization imprint when graphene is deposited, from *P*^(−)^ for clean samples to *P*^(+)^ for samples with carbon deposited, or the enhanced photodissociation of the surface in presence of carbon and high intensity synchrotron radiation soft X-ray beam.

## Conflicts of interest

There are no conflicts to declare.

## Supplementary Material

RA-010-C9RA09131A-s001
