# Supplementary material for: Resistance hysteresis correlated with synchrotron radiation surface studies in atomic sp2 layers of carbon synthesized on ferroelectric (001) lead zirconate titanate in an ultrahigh vacuum
Source: RSC Adv. 2020 Jan 8;10(3):1522–34. doi: 10.1039/c9ra09131a (PMC9047335; doi:10.1039/c9ra09131a)
Supplement: RA-010-C9RA09131A-s001 [file RA-010-C9RA09131A-s001.pdf]

Electronic Supplementary Material (ESI) for RSC Advances.
This journal is © The Royal Society of Chemistry 2020

Electronic Supplementary Information

Resistance hysteresis correlated with synchrotron radiation surface studies in atomic *sp*[2] layers of carbon synthesized on ferroelectric (001) lead zirconate titanate in ultrahigh vacuum

Nicoleta Georgiana Apostol,[a] Daniel Lizzit,[b] George Adrian Lungu,[a] Paolo Lacovig,[b] Cristina Florentina Chirilă,[a] Lucian Pintilie,[a] Silvano Lizzit [b] and Cristian Mihai Teodorescu*[a]

[a.] *National Institute of Materials Physics, Atomiștilor 405A, 077125 Măgurele – Ilfov, Romania. E-mail teodorescu@infim.ro*
[b.] *Elettra Sincrotrone Trieste, Strada Statale 14 – km 163,5, Area Science Park, 34149 Basovizza, Trieste, Italy.*
**corresponding author, e-mail teodorescu@infim.ro*

1. Estimate of square resistances and mobilities

The average resistances (mediated over the V_G cycles) are 216.5 Ω, 384.6 Ω, and 212.0 Ω for 0.64 ML, 2.93 ML, and 7.55 ML deposited carbon, respectively. This non-linear behaviour of the resistance as function of the carbon coverage can be fit by taking into account the amount of carbon atoms forming in-plane sp^2 bonds, which is the product between the proportion $p(sp^2)$ and the carbon coverage (see Table 1 and Fig. S1). This fit suggests the existence of a parasitic contact resistance between the Pt electrodes and the surface of PZT(001), including when carbon is deposited on top. The empirically derived almost linear dependence of the resistance with the total amount of carbon which exhibit in-plane sp^2 bonds can qualitatively be connected with the long stripes of equal V_z voltage measured on a large (microscopic) scale and represented in Fig. 5(a). The fitting for the resistance value is represented in Fig. S1 and it follows that the resistance which would correspond to a pure graphene-like layer would be 154.4 Ω. The square resistivity of a 1 ML carbon film, taking into account that the electrodes are separated by 2 mm and their parallel edges are 1 mm wide (see Fig. S2), will be half of the above value, i. e. $R_\blacksquare \approx 77.2$ Ω. This surface resistance may be used to derive the moblity of the graphene-like layer:

$$\mu = \frac{1}{R_\blacksquare e \delta n_S} \qquad \text{(S1-1)}$$

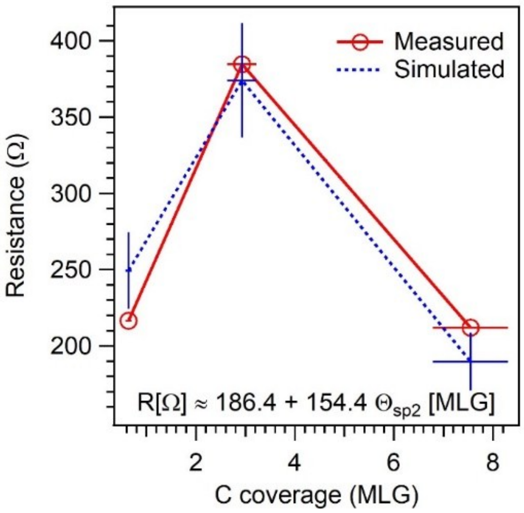

Fig. S1 Red symbols: source-drain resistance measured between the same Pt electrodes for several amounts of carbon deposited. Blue symbols: a fit considering this resistance proportional to the amount of carbon atoms forming in-plane sp^2 bonds, derived from NEXAFS spectroscopy.

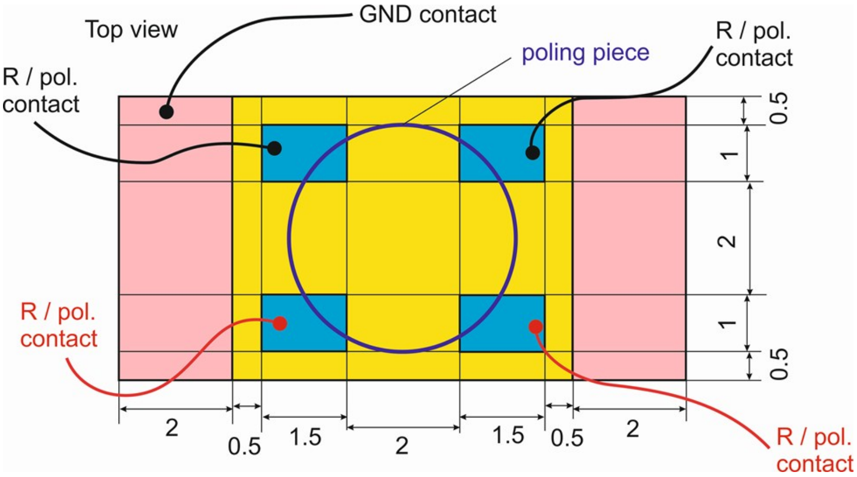

Fig. S2. A top view of the sample. The red contacts were the ones for which the actual data are reported, for all thicknesses of carbon layers. The distance between electrodes is 2 mm, and their parallel edges are 1 mm wide.

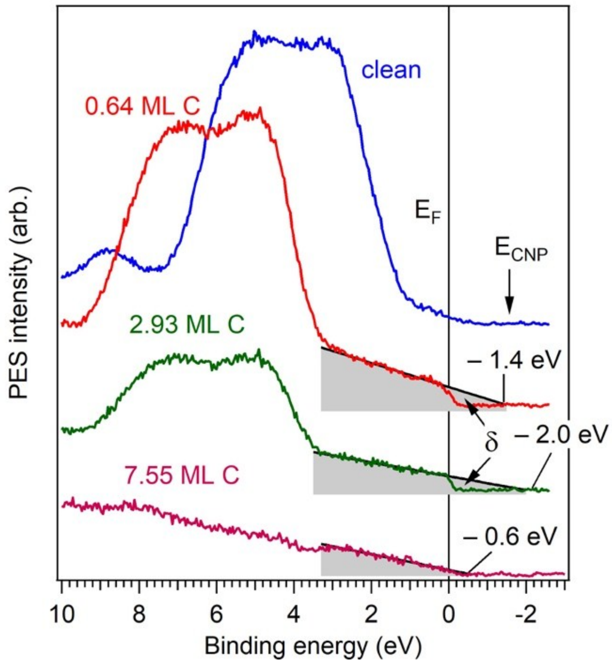

Fig. S3 The angle integrated valence band photoemission spectra recorded with synchrotron radiation (photon energy 260 eV). The Fermi level is located in the valence band. The δ parameter may be estimated from the position of the Fermi level with respect to the change neutrality point (Eq. (1) from the main text).

In the above formula, n_S is the surface density of atoms in graphene $n_S = 1/(2.62\ \text{Å})^2 \approx 3.82 \times 10^{19}\ \text{m}^{-2}$ and δ is the amount of conduction charge in the graphene layer corresponding to a C atom (δn_S is the surface density of electrons or holes). δ may be estimated from valence band spectra (Fig. S3). The density of states for a 2D system with particles described by massless Dirac fermions is linear with energy, and this is visible for all valence band spectra from Fig. S3. The δ parameter may be estimated with eq. (1) from the main text and yields 0.038 for the 0.64 ML film, 0.077 for the 2.93 ML film and 0.007 for the 7.55 ML film. Introducing in eq. (SI-1), the corresponding mobilities are 574, 276 and 3069 $\text{cm}^2\text{V}^{-1}\text{s}^{-1}$, respectively. Except for a huge mobility of $7 \times 10^4\ \text{cm}^2\text{V}^{-1}\text{s}^{-1}$ reported in an early work,[14] most values obtained for mobility for the experiments with transferred graphene range between a few hundreds and 1300 $\text{cm}^2\text{V}^{-1}\text{s}^{-1}$.[1,17] Thus, although imperfect as seen from the STM images, the surface transport properties of the Csp^2 are not significantly different to most of the transferred graphene layers studied so far.

2. Dependence of the adjustable tip-sample distance and voltage on the piezo scanner V_z on local sample biases on the surface

The operating scheme of a scanning tunnelling microscope in constant current mode is represented in Fig. 1. The piezo scanner is activated by an applied voltage along it, V_z, such as to adjust with fine precision the tip-sample distance *d* by keeping the tunnelling current I_t constant with a feedback loop.[S1] In this mode, the STM image is in fact a map of the read values of V_z along the scan, by using a piezo conversion factor a defined as the relative elongation divided by the applied electric field, in units of length/voltage. The tunnelling voltage V_t is also constant during a scan. Therefore, if some bias is present on the sample surface, such as in the case of free ferroelectric surfaces,[35] the necessity to keep I_t constant will be translated in a change of the distance *d* and hence the applied V_z on the piezo scanner.

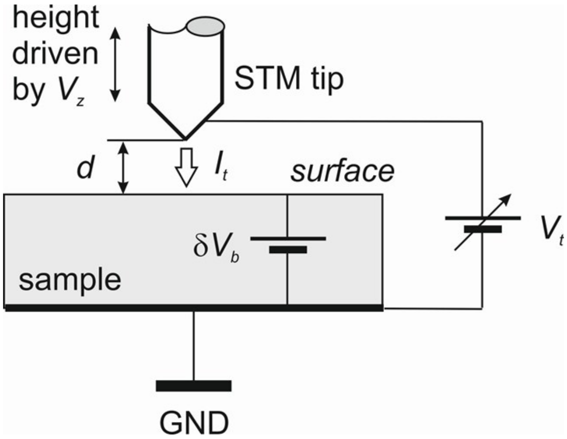

Fig. S3. Schematics of an STM scan, with all the voltages involved in the measurement.

A connection between the tunnelling current and the distance d may be traced starting with the expression for the tunnelling through a potential barrier of width d and height ($V_0 - V_t$).[S1] One considers:

$$I_t(V_t,d) \approx G_0 V_t \exp\{-2\kappa(V_t)d\} \tag{S2–1}$$

with G_0 having the dimensions of a conductance and where

$$\kappa(V_t) = \frac{\{2me(V_0 - V_t)\}^{1/2}}{\hbar} \tag{S2–2}$$

and obtains simply:

$$d = \frac{\hbar}{2\{2me(V_0 - V_t)\}^{1/2}} \log\frac{V_t}{W} \tag{S2–3}$$

where

$$W = \frac{I_t}{G_0} \tag{S2–4}$$

κ is on the order of 1 Å^{-1}, *d* on the order of 1 nm, therefore $\exp\{-2\kappa(V_t)d\} \sim e^{-20} \sim 2 \times 10^{-9}$. G_0 is therefore on the order of 1 Ω^{-1} and *W* is on the order of some nV.

In 'practical' units:

$$d[\mathrm{nm}] = \frac{0.09765}{\{V_0[\mathrm{V}] - V_t[\mathrm{V}]\}^{1/2}} \log \frac{V_t}{W}$$

(S2–5)

Some evaluations of eq. (5) are given in Fig. S4. One observes that the dependence is almost linear for 0.5 < V_t < 2.5. Thus, V_z also may be considered linear in V_t.

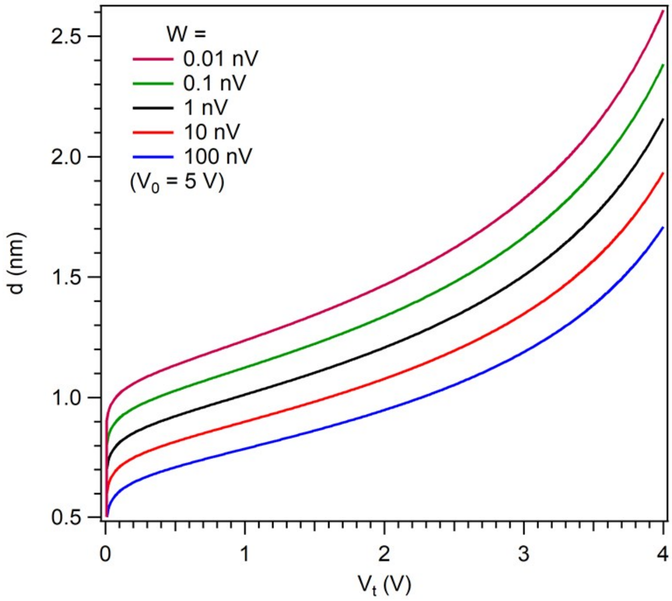

Fig. S4. Dependence on the tip-sample distance for a constant tunnelling current on the tip-sample voltage. The tip current is proportional to the parameter *W*, see eq. (S4).

One may then apply a theory of linear response for variations of V_t implied by the presence of a surface bias voltage δV_b. The constancy of the tunnelling current means that in presence of the surface voltage bias δV_b the distance will vary by δd.

$$I_t(V_t, d) = I_t(V_t - \delta V_b, d - \delta d)$$

(S2–6)

Developing in the first order:

$$I_t(V_t - \delta V_b, d - \delta d) \approx I_t(V_t, d) - \left(\frac{\partial I_t}{\partial V_t}\right)_d \delta V_b - \left(\frac{\partial I_t}{\partial d}\right)_{V_t} \delta d$$

(S2–7)

We take into account the variation of *d* by means of V_z:

$$\delta d = \alpha \delta V_z$$

(S2–8)

where α = 1.3 nm/V for the Aarhus 150 STM system, value communicated by the manufacturer.
It follows

$$\delta V_b = -\alpha \delta V_z \left(\frac{\partial I_t}{\partial d}\right)_{V_t} \left(\frac{\partial I_t}{\partial V_t}\right)_d^{-1}$$

(S2–9)

Introducing eqs. (S2–1, S2–2), after derivation and some algebra:

$$\delta V_b = \alpha \delta V_z \frac{\hbar^2 \kappa^2}{d \cdot m \cdot e} = \frac{2\alpha (V_0 - V_t)}{d + \frac{\hbar}{(2me)^{1/2}} \frac{(V_0 - V_t)^{1/2}}{V_t}} \delta V_z \equiv f \delta V_z$$

(S2–10)

where the factor f can be written in practical units:

$$f \approx \frac{2\alpha[nm\,V^{-1}](V_0 - V_t)[V]}{d[nm] + 0.1953\frac{(V_0[V] - V_t[V])^{1/2}}{V_t[V]}}$$

(S2–11)

The main problem in applying quantitatively this method is that it is difficult to estimate the 'normal' measuring distance d or the value of V_0. But if one estimates $V_0 \approx 5$ V, $V_t \approx 0.2$ V and $d \approx 1$ nm, it follows $\delta V_b \sim \delta V_z$. This factor is quite critically depending on the tip voltage, see Fig. S5.

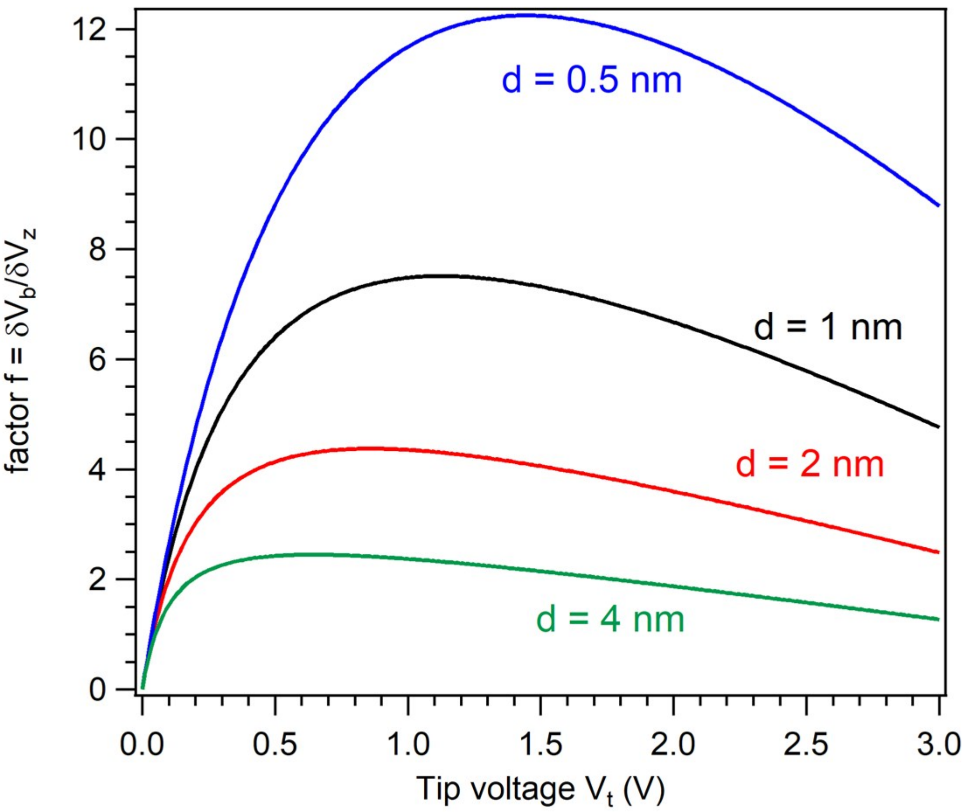

Fig. S5. Factor between the surface bias voltage and the voltage on the piezo scanner, eq. (S11), for several values of the tip-sample distance where the tip would be placed in absence of the bias voltage, for $\alpha = 1.3$ nm V^{-1} and $V_0 = 5$ V.

3. XPS measurements using a laboratory X-ray source

X-ray photoelectron spectroscopy was performed on 50 nm thick $PbZr_{0.2}Ti_{0.8}O_3$ (PZT) on the CoSMoS – Combined Spectroscopy and Microscopy on Surfaces – facility, using a conventional X ray gun with Al K_α excitation (1486.74 eV). The sample was investigated after a cleaning procedure[S2,42] and then after the C deposition. There is no surface reduction or release of metal Pb. The Zr/(Zr + Ti) ratio is 0.21. The carbon coverage was slightly different than the lowest carbon coverage from the synchrotron radiation experiments, it was estimated at about 1.0 – 1.1 ML obtained by two methods: (a) the Pb $4f_{7/2}$ peak attenuation and (b) from the C:Pb line integral intensity, normalized by the XPS atomic sensitivity factors. The significance of the high BE component is discussed in the text. There was no need in this case to introduce a low BE component. Thus, there was no need to introduce a component responsible for cationic

vacancies. This results also from the surface compositional analysis, yielding $Pb_{1.1}Zr_{0.21}Ti_{0.79}O_{2.15}$. Also, the shift introduced by carbon deposition is lower in this case with respect to the synchrotron radiation experiment (0.67 eV) which may indicate that in this case the initial state is partly $P^{(+)}$ polarized, in line with the detected oxygen vacancies near surface, needed to generate electrons for compensation of the depolarization field.

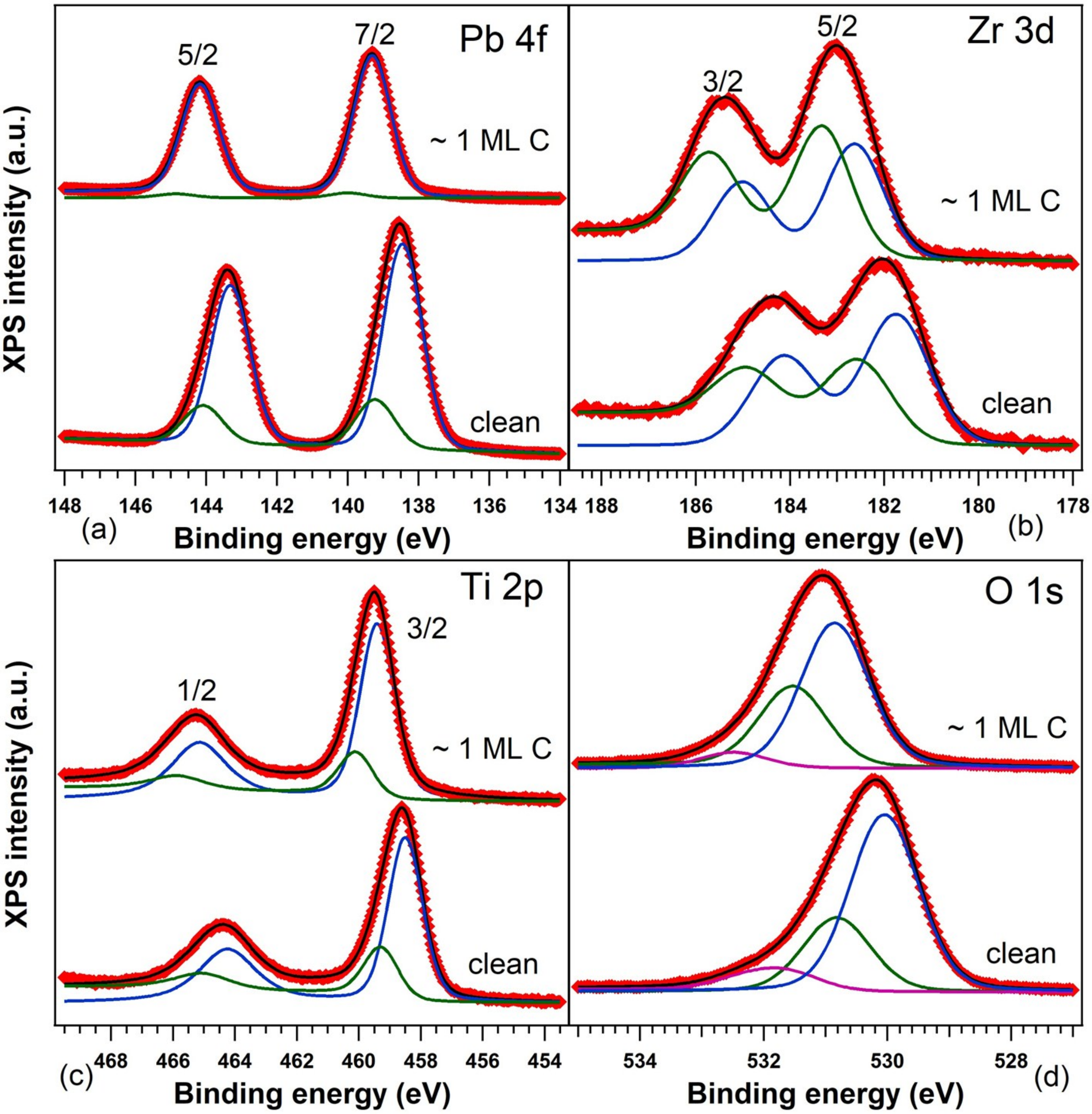

Fig. S6. X-ray photoelectron spectra obtained using a laboratory source: (a) Pb 4f, (b) Zr 3d, (c) Ti 2p and (d) O 1s, with 'deconvolution' using Voigt doublets for (a–c) and Voigt singlets for (d). The pass energy of the electron energy analyzer was 30 eV.

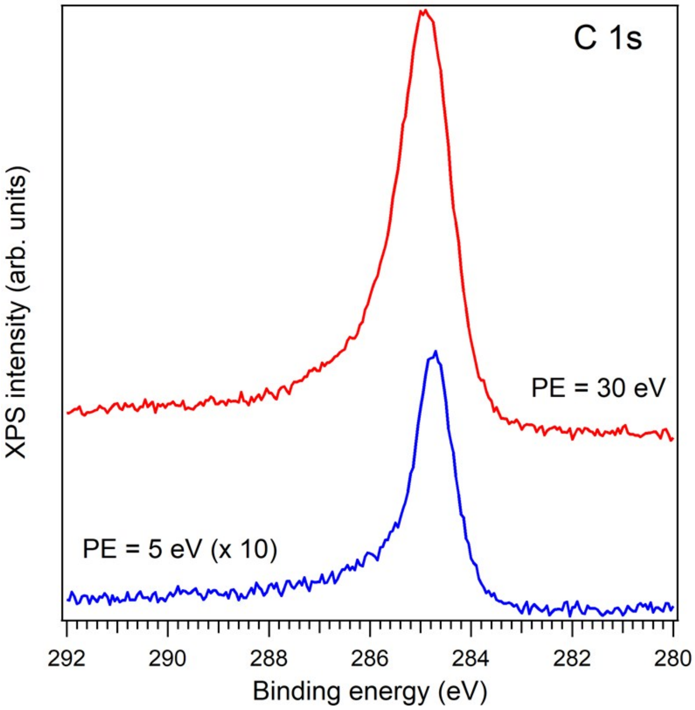

Fig. S7. C 1s spectra obtained after deposition of ~ 1 ML carbon on PZT(001), with two pass energies of the analyzer, 30 eV in order to compare with the other core levels from Fig. S6 and 5 eV to yield a spectrum with good resolution.

Reference:

S1. C. Julian Chen, *Introduction to Scanning tunnelling Microscopy*, Oxford University Press, Oxford, 1993.
S2. I. Krug, N. Barrett, A. Petraru, A. Locatelli, T.O. Mentes, M.A. Niño, K. Rahmanizadeh, G. Bihlmayer, and C.M. Schneider, Extrinsic screening of ferroelectric domains in $Pb(Zr_{0.48}Ti_{0.52})O_3$, Appl. Phys. Lett. 97, 222903(1-3) (2010).
Ref. 42, listed in the main text.
